# Arrhythmia Disease Diagnosis Based on ECG Time–Frequency Domain Fusion and Convolutional Neural Network

**DOI:** 10.1109/JTEHM.2022.3232791

**Published:** 2022-12-28

**Authors:** Bocheng Wang, Guorong Chen, Lu Rong, Yuchuan Liu, Anning Yu, Xiaohui He, Tingting Wen, Yixuan Zhang, Biaobiao Hu

**Affiliations:** School of Intelligent Technology and EngineeringChongqing University of Science and Technology 66564 Chongqing 401331 China; Chongqing Vocational Institute of Engineering 519527 Chongqing 402260 China

**Keywords:** Time–frequency domain fusion, convolutional neural networks, ECG diagnosis

## Abstract

Electrocardiogram (ECG) signals are often used to diagnose cardiac status. However, most of the existing ECG diagnostic methods only use the time-domain information, resulting in some obviously lesion information in frequency-domain of ECG signals are not being fully utilized. Therefore, we propose a method to fuse the time and frequency domain information in ECG signals by convolutional neural network (CNN). First, we adapt multi-scale wavelet decomposition to filter the ECG signal; Then, R-wave localization is used to segment each individual heartbeat cycle; And then, the frequency domain information of this heartbeat cycle is extracted via fast Fourier transform. Finally, the temporal information is spliced with the frequency domain information and input to the neural network for classification. The experimental results show that the proposed method has the highest recognition accuracy (99.43%) of ECG singles compared with state-of-the-art methods. Clinical and Translational Impact Statement— The proposed ECG classification method provides an effective solution for ECG interrogation to quickly diagnose the presence of arrhythmia in a patient from the ECG signal. It can increase the efficiency of the interrogating physician by aiding diagnosis.

## I. Introduction

Arrhythmia, a cardiovascular disease (CVD), threat to human life and health [1], [2], [3], [4], [5], and it is the leading cause of death and disability occurrence worldwide, along with cerebrovascular disease [6]. CVD tops the list of causes of premature human death in more than seventy countries worldwide [7], [8]. Early detection of patients with critical arrhythmia symptoms can be of great help in treating patients with cardiovascular disease and avoiding more serious consequences [9], therefore, the diagnosis, prevention, ambulance and treatment of cardiac diseases are crucial issues in the field of cardiology.

The ECG signal has a more intuitive regularity. It represents the electrical activity of the heart [10]. The signal contains a great deal of biological health information [11]. The ECG signal can be used to diagnose whether the test subject has ventricular atrial hypertrophy, myocardial ischemia and arrhythmias [12], while the ECG signal is easier to be detected than other bioelectrical signals. Therefore, ECG signals became one of the first biological signals studied by humans and applied in medical clinics [13]. Many studies have been conducted on the recognition of heart health status based on ECG signals [14], [15], [16], [17], [18]. The waves in the ECG wave cluster are named in alphabetical order and are P, Q, R, S, T, and U. The shape, amplitude, and duration of these waves indicate different information about the state of the heart. The P wave is generated by depolarization of the atria, and the QRS wave group is generated by depolarization of the ventricles. The T and U waves arise from repolarization of the ventricles. Traditionally, the ECG diagnosis is made by the specialist by observing the information of wave clusters in the patient’s ECG. For example, when the duration of the QRS wave cluster is too long, the patient may have a bundle branch conduction block [19]. When abnormal changes are produced in the ST wave cluster, it may indicate the presence of diseases such as myocardial infarction or angina in the patient [20]. However, traditional ECG diagnosis methods rely on physician expertise and require a complex feature extraction process [21]. Therefore, more deep learning methods are used for the classification of ECG signals. Cui et al. [22] used convolutional neural network (CNN) and support vector machine (SVM) to classify ECG signals, thereby to diagnosis the status of heart. Zeng et al. [23] performed variational pattern decomposition (VMD) of the ECG signal and fed it into an artificial neural network (ANN) to achieve the identification of five heartbeat types. Jikuo et al. [24] proposed a novel convolutional neural network with a non-local convolutional block attention module (NCBAM) for ECG signal classification of single heartbeat cycles. Subasi et al. [25] used an iterative relief and neighborhood component analysis (NCA) based feature selection approach for ECG signals. The final features were fed into a deep neural network (DNN) to obtain good diagnostic results. Sinha et al. [26] extracted spectral coherence index (SCI) and phase coherence index (PCI) from the spectrum of 12-lead ECG signals, then fed the features into a framework integrated with SVM classifier for myocardial infarction diagnosis of the heart. Amrani et al. [27] used very deep convolutional neural network (VDCNN) for feature extraction of ECG signals and reduced the model computation by feature fusion and finally classified the ECG signals with better results and generalization ability.

These studies use various methods to extract features from ECG signals. Finally, the extracted features are used to diagnose the heart health status through classifiers or neural networks. However, these methods are basically based on the time domain or frequency domain information of the ECG signal, without considering the information in both time and frequency domains of the ECG signal. Therefore, a method based on convolutional neural network to fuse the time and frequency domain information of ECG signals for heart status diagnosis is proposed. Firstly, the filtering of ECG signals is completed by wavelet decomposition. Next, the individual heartbeat cycles are extracted by the R-wave position of each heartbeat cycle. A fast Fourier transform is performed on the extracted heartbeat signal to obtain its frequency domain information. The time domain signals are spliced with the frequency domain signals and then fed into a one-dimensional convolutional neural network (1D-CNN) for fusion and feature extraction. Finally, the feature matrix is fed into a fully connected neural network to complete the classification.

The main contributions of this paper are as follows:

1. A novel heartbeat cycle localization algorithm is proposed. The algorithm can simultaneously calibrate the R-wave crest positions of normal ECG signals and diseased ECG signals. This allows each heartbeat cycle in the ECG signal to be better segmented, thus improving the accuracy of subsequent classification.
2. The fast Fourier transform is introduced to extract the frequency domain signal of the heartbeat cycle. The performance of single heartbeat cycle in both time and frequency domain perspectives is fused by CNN to classify ECG signals in an integrated manner. The classification accuracy of single-heartbeat cycle ECG signals is improved to exceed the latest methods.
3. A shallow layer CNN is used to keep the size of the model small and to classify ECG signals with limited hardware resources, and we use a convolutional filter with a large sensory field and a small step size. This results in an improved quality of feature extraction with a guaranteed shallow layer network. This model is suitable to be deployed in small devices, which provides an idea for miniaturization of ECG signal-assisted diagnostic devices.

## II. Overview

### A. Automatic Diagnosis of ECG Signals

Traditional diagnosis of heart disease by ECG signals requires a large amount of ECG monitoring data and a highly qualified physician. Automatic classification of ECG signals can save medical resources and increase efficiency. Numerous studies have been conducted on the automatic classification of ECG signals. Such research mainly includes the feature extraction and classification of ECG signals.

Eigenvalue extraction of the ECG signal is to extract some of the key information of the ECG signal that contains the heart activity, so that the ECG signal can be represented in a lower dimension. This information can be used to diagnose ECG signals with high accuracy with less information. The traditional feature extraction methods are pattern recognition methods, among which the main methods are wavelet transform [28], frequency analysis, principal component analysis [29], independent component analysis [30], etc. In recent years, with the development of artificial intelligence technology, a large number of deep learning methods have been used for feature information extraction of ECG signals, and the main methods include CNN [31], multilayer perceptron (MLP), recurrent neural network (RNN), deep belief network (DBN) [32], long and short term memory network (LSTM) [33], gated recurrent unit (GRU) and bidirectional recurrent neural network (BRNN), etc [34].

In the classification stage of ECG signal classification is mainly done by various classifiers, such as conditional random field (CRF), support vector machine, dynamic Bayesian network (DBN), neural network and decision tree (DT). The increasing popularity of deep learning approaches in recent years, along with the existence of several authoritative ECG signal datasets online, has led a large number of scholars and research teams to try to build various neural network models to solve the automatic ECG signal classification problem.

### B. ECG Frequency Domain Information Extraction

The ECG signal is a time-series signal, which is essentially a voltage signal that changes over time. In the past, the traditional ECG diagnosis in hospitals was made by observing the ECG drawn from the time-series ECG signal, and the diagnosis was based on whether the ECG drawn from the ECG signal was a typical shape of a healthy ECG. If the shape deviates significantly from a healthy ECG, the ECG is considered to be diseased. However, there are many frequency domain features in medicine that are associated with certain diseases. When arrhythmias occur, QRS wave groups change significantly, leading to abnormal changes in the high-frequency components [35]. It follows that the frequency domain features of the ECG signal can also contain a portion of information about the pathology that may be difficult to be observed in the time domain. Therefore, the extraction of frequency-domain information from ECG signals can be of great help in discovering lesion information that is difficult to be observed in the time domain. Among the methods for frequency domain feature extraction of ECG signals, the most famous ones are fast Fourier transform (FFT) and wavelet transform (WT) [36]. The fast Fourier transform turns the time-series signal into the distribution of each frequency in the frequency domain by means of an infinitely long trigonometric basis. Its transform represents the maximum amplitude of the signal in each frequency, but it is not possible to determine the generation time of the signal in these frequencies. In contrast, the wavelet transform uses a finite-length wavelet basis, and the corresponding frequencies and corresponding times are controlled by the scale and translation of the wavelets, and the time-frequency spectrum of the signal can be obtained. The spectrum contains the distribution of each frequency on the time axis. In addition to extracting the time-frequency domain information of the signal, the wavelet transform can also be used for R-wave localization of ECG signals [37]. In this paper, the wavelet transform is used to filter the original ECG signal and R-wave localization, and the fast Fourier transform is used to extract the frequency domain information of the ECG signal.

### C. Convolutional Neural Networks

A convolutional neural network (CNN) is a deep learning algorithm generally used for feature extraction of information. It is widely used in image processing. Amrani et al. [38], [39], [40], [41] used CNN with other algorithms for the processing of Synthetic aperture radar images. A CNN generally consists of three elements: a convolutional layer, a pooling layer and a fully connected layer, each layer takes input from the previous layer and outputs the processed result to the next layer. The convolutional and pooling layers in the network are generally combined before and after to complete feature extraction and data dimensionality reduction. The data is convolved and pooled several times to obtain a feature matrix. The final result is obtained by feeding it to the fully connected layer. Through training, the parameters in the network are continuously optimized so that the error between the prediction result and the label is as small as possible.

## III. Methodology

In this study, the frequency domain information of the ECG signal is extracted via FFT. The time and frequency domain information of ECG signal is fused by CNN to classify. In practical applications, the ECG signals collected in real time are not calibrated for the R-wave wave crest position. Therefore, R-wave localization needs to be performed after the human ECG signal is acquired. Subsequently, the localization information is used to input each complete heartbeat cycle into the model for diagnosis. Therefore, the research route is divided into four parts: signal preprocessing, R-wave localization, frequency domain information extraction, and ECG diagnostic model construction.

### A. Signal Pre-Processing

The ECG signal is a relatively weak signal, with low frequency and amplitude. These characteristics make the ECG signal to be easily disturbed by various noises during the acquisition process. Such as the acquisition process, the patch electrode on the body sliding electrode position changes and the body’s breathing caused by the movement. Both of these will cause the ECG signal to produce a certain baseline drift, so that the regularity of the ECG signal with time related changes. The variation pattern is very similar to that of a low frequency sinusoidal function. The involuntary fibrillation of the body’s muscles can also have an effect on the ECG signal. This interference is known as myoelectric interference, and it is often high frequency and irregular. The environment where the acquisition device is powered can produce electromagnetic interference to the device, which is hard to avoid. Myoelectric and electromagnetic interference tend to mask small but critical information in the ECG signal.

The ECG signal is mixed with high-frequency, low-amplitude noise. These noises mask some waveforms of smaller amplitude in the ECG signal. Such noise interference can have a significant impact on the subsequent R-wave localization and frequency domain analysis, and also affect the final classification accuracy of the model. Therefore, the original ECG signal should be filtered before the subsequent work is performed.

Since the noise and signal are mixed together, but they have large differences in the frequency domain, the information of different frequencies in the signal can be decomposed by multi-scale wavelet decomposition. At the same time, wavelet transform has the feature of considering both time domain and frequency domain information of the signal, so it is suitable for processing ECG signals which are non-stationary signals. The mathematical expression of continuous wavelet transform (CWT) is shown by the following equation (1).

\begin{equation*} W T(\alpha, \tau)=\frac {1}{\sqrt {\alpha }} \int _{-\infty }^{\infty } f(\mathrm {t}) \psi \left ({\frac {\mathrm {t}-\tau }{\alpha }}\right) \mathrm {dt}\tag{1}\end{equation*}

where, $W T(\alpha, \tau)$ is the result after wavelet transform, $f(t)$ is the signal for wavelet transform, $t$ is the time, $\psi (\mathrm {t})$ is the wavelet mother function, $\alpha $ is the scale of wavelet, and $\tau $ is the amount of wavelet translation. However, in CWT, the scale and translation of wavelets are continuously varied. Continuous wavelet transform of ECG signals can lead to results with excessive redundant information. This has a negative impact on the denoising and signal reconstruction of ECG signals. In contrast, the discrete wavelet transform(DWT) is more suitable to be used for the processing of ECG signals [42].

The discrete wavelet transform needs to discrete transform the scale $a$ of the wavelet with the translation $\tau $, so that $\alpha =\alpha _{0}^{\mathrm {m}}, \tau =n \alpha _{0}^{m}, m, n \in Z$. The DWT is calculated by the following equation (2).

\begin{equation*} {DWT}(m, n)=\frac {1}{\sqrt {\alpha _{0}^{m}}} \int _{-\infty }^{\infty } f(t)^{*} \psi \left ({\frac {t-n \alpha _{0}^{m}}{\alpha _{0}^{m}}}\right) dt\tag{2}\end{equation*}

If $f(n)$ is a discrete signal, the wavelet transform of it is given by equation (3)

\begin{equation*} f(n)=A[f(n)]+D[f(n)] \tag{3}\end{equation*}

where $A$ is the low frequency component of $f(n)$ decomposed by the wavelet transform and $D$ is the high frequency component of the decomposition. In the decomposition of the signal using wavelet transform only the low frequency components of the signal are decomposed, while wavelet decomposition has excellent performance in the decomposition of the low frequency part [43].

**FIGURE 1.**
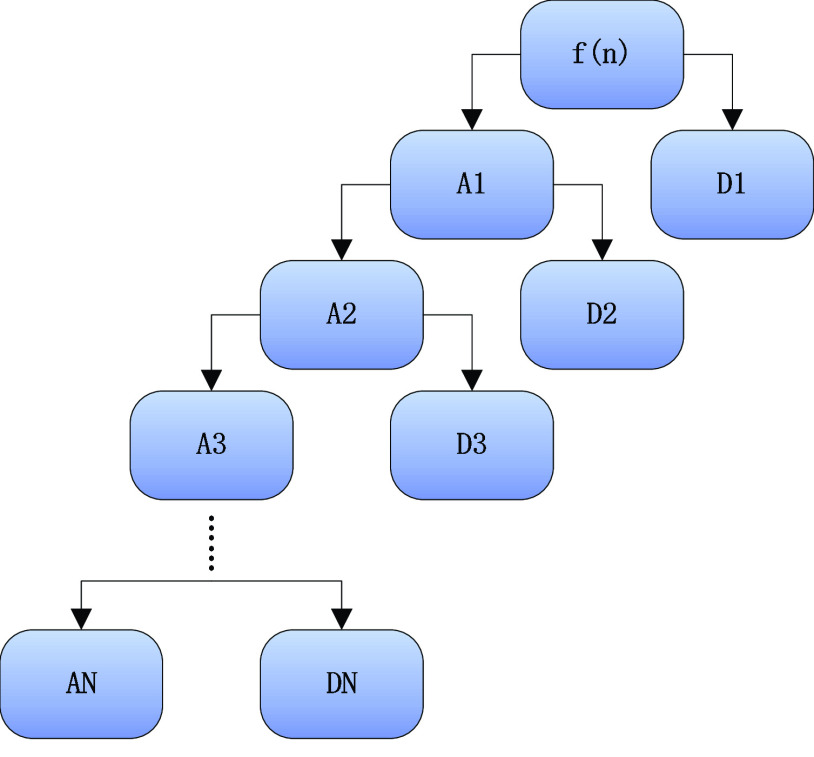
N-scale wavelet decomposition schematic.

In this study, we refer to the filtering method in the paper [44] and use wavelet bases to decompose the signal at $N$ scales whose process is shown in Figure 1.

The original signal is decomposed and the two highest frequency components are zeroed to filter out the high frequency interference in the original signal. The low amplitude portion of the decomposed other high frequency components is derived from high frequency low amplitude noise, and the wavelet coefficients in the other high frequency components that are smaller than the threshold can be set to zero by setting a threshold $\lambda $ using soft threshold filtering. Wavelet reconstruction of the processed *D1* to *DN* with *AN* result in a noise-removed ECG signal. The threshold $\lambda $ for soft threshold filtering is calculated as in equation (4).

\begin{equation*} \lambda =\frac {median|D|}{0.6745 * \sqrt {2 * \log (C)}} \tag{4}\end{equation*}

where $D$ is the processed wavelet coefficient and $C$ is the signal length.

### B. R-Wave Localization

**FIGURE 2.**
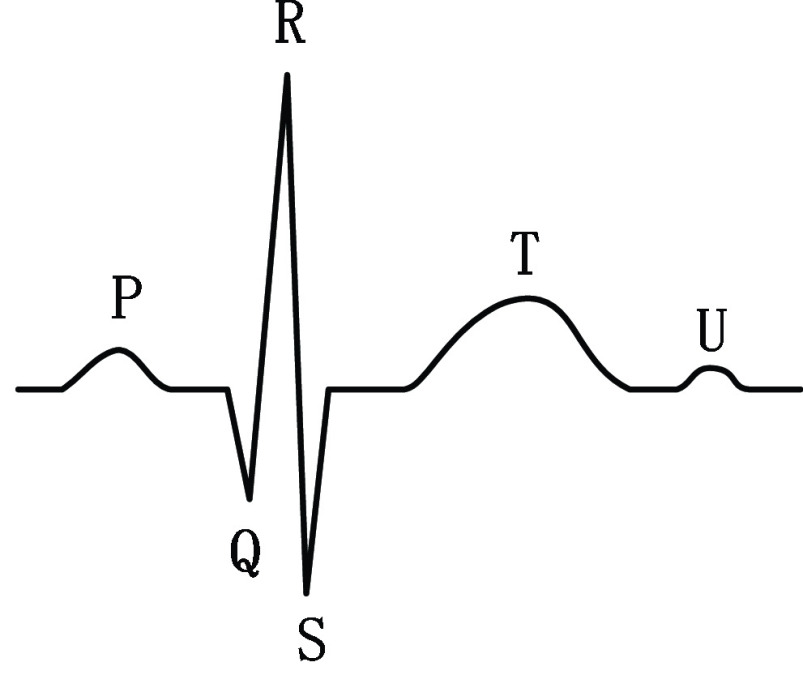
ECG waveform.

The classification of the ECG signal in this study is done based on each independent heartbeat cycle. Each QRS fluctuation on the ECG represents one heartbeat, as shown in Figure 2, and one heartbeat can be located by capturing the peaks of the R waves. The labeled data from the MIT-BIH arrhythmia dataset was used in the model training process, and the location information of the R-wave crest in the ECG signal was directly obtained, and each heartbeat cycle of the ECG signal was segmented by this location information. However, in practical applications, the R-wave positions of the ECG signal are not labeled, so the R-wave positions in the ECG signal need to be located before using the model in this study to classify the ECG signal in order to segment each individual heartbeat cycle.

The traditional R-wave identification method takes advantage of this property by setting a threshold to identify the first-order differential data of ECG signals, but this method is only accurate for typical healthy ECG signals, but the accuracy of this method is significantly reduced for diseased signals. This is due to the fact that the slope of the R-wave decreases in some pathological signals, while many wavelets with slopes greater than the R-wave are generated elsewhere.

It was found that the energy of the QRS wave is mainly concentrated in the third scale when the wavelet transform of the ECG signal is performed, and the R-wave transform on this scale produces a pair of mode maxima, i.e., a positive maxima-negative minima pair, and the moment of R-wave peak taking corresponds to the over-zero point between the maxima and minima on this scale [45]. At the same time, the characteristics of the QRS wave positions of the lesion signal after wavelet transform at this scale are more similar to those of a typical healthy ECG signal, and most of the interference of the lesion waveform is filtered out. Using such a method has a better performance for segmenting the heartbeat cycle of the lesioned signal.

**FIGURE 3.**
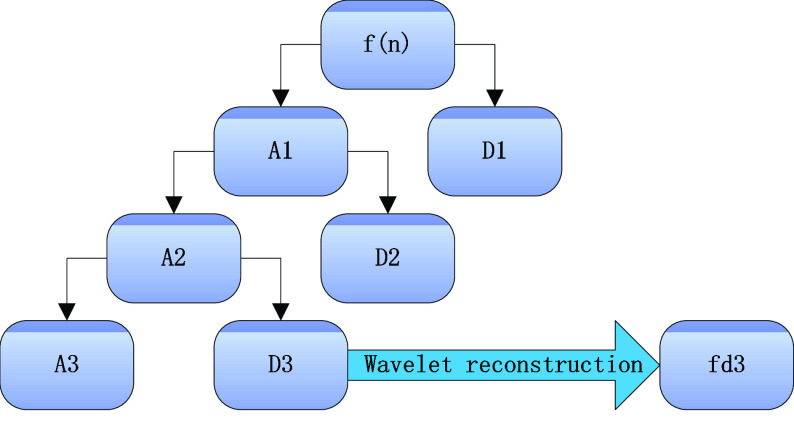
Wavelet third scale decomposition reconstruction.

In this paper, three scales of decomposition of ECG signals are performed using db3 wavelet basis. Then the high frequency component of the third scale, *D3*, is reconstructed to obtain *fd3*. *fd3* is used for subsequent R-wave localization. The process is shown in Figure 3.

**FIGURE 4.**
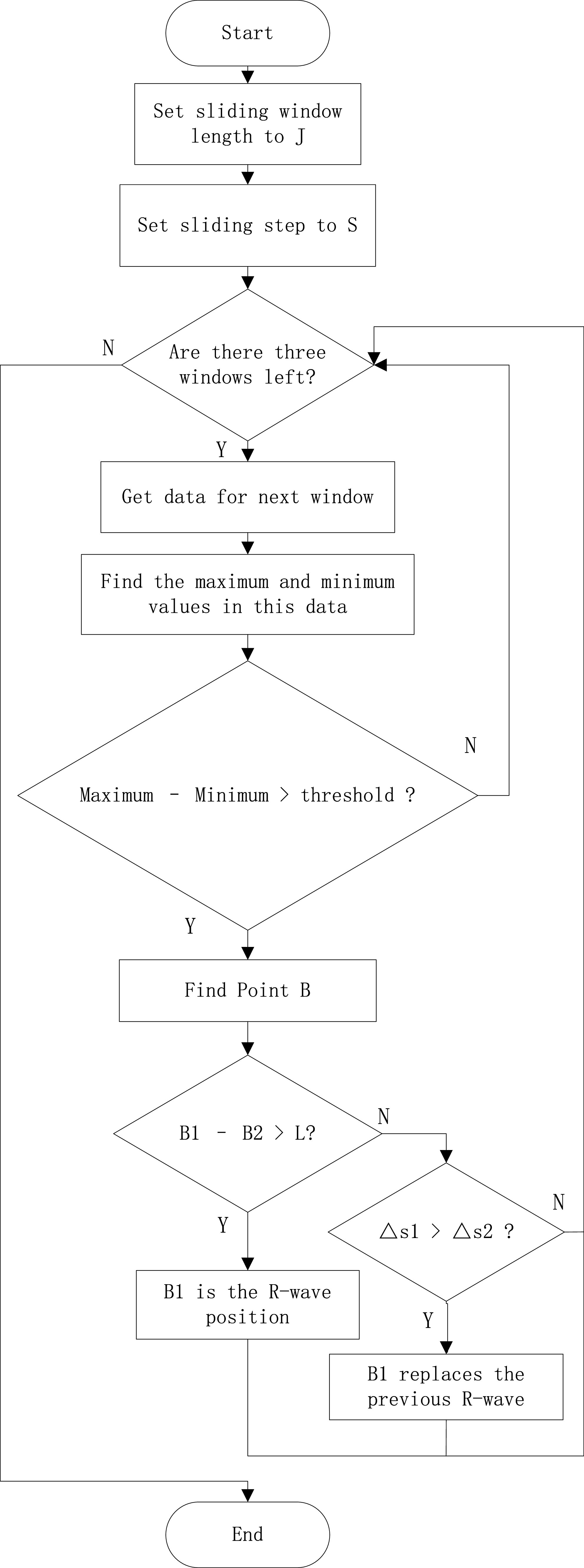
R-wave localization algorithm flow.

However, it has been found experimentally that the R-wave crest points do not always strictly correspond to the over-zero points of the maxima and minima at that scale, and there is often some offset. In this study, the extreme values generated by the QRS wave group in fd3 are located by sliding window and setting a threshold. Let the location of the extreme value be point A. Set the interval width to $2K$. Find the value of the ECG signal $f(n)$ with the largest difference from the signal mean in the interval around the position of point A. Set it as $\Delta $ *s1*, set it as $\Delta $ *s2* as the previous $\Delta $ *s1* value, set the position of this value as point *B1*, and set *B2* as the position of the previous *B1* point. The reason for looking for the value with the greatest difference from the mean value of the signal instead of the maximum value is that in some lesions the R-wave is downward, and it is the trough that should be looked for instead of the peak. By looking for the absolute value, it is possible to find the corresponding point in both cases. Considering the time difference between two heartbeats, the distance between the position located this time and the previous position is calculated. When the distance is less than $L$, the point that has a larger value in the ECG signal than the mean value of the signal is taken, and vice versa, a new heartbeat is considered, and the algorithm flow is shown in Figure 4.

### C. Frequency Domain Information Extraction

Since some cardiovascular lesions are difficult to be identified in the time domain representation of the ECG signal, but are easily detected in the high-frequency part of the frequency domain. Therefore, the frequency domain information of the ECG signal is extracted and its time series signal is spliced, and then fed into a one-dimensional convolutional network for recognition and classification to improve the accuracy of recognition.

Based on the results of R-wave localization, the R-wave position with the first n-1 and the next $2n$ data, a total of $3n$ data, is extracted into a heartbeat cycle signal to be identified. The frequency domain information of this heartbeat signal is obtained by performing FFT of this signal with a frequency of sampling frequency $F$ using the FFT. Since the transformed spectrum is centrosymmetric, generally only the first half is taken to obtain the maximum amplitude of each frequency from 0Hz to F/2Hz. The ECG signal is stitched back and forth with the frequency domain information to form the data to be identified with a length of 3n $+F$/2

### D. ECG Diagnostic Model Building

With the development of artificial intelligence technology, more and more scholars have used deep learning techniques for feature recognition of ECG signals [46], [47], [48], [49], [50]. CNN generally have both feature extraction and classification functions. The data is input through the input layer, then the feature extraction is performed through the convolutional and pooling layers, and finally the classification results are output using the fully connected neural network. The training process of the network model is to update the values in the convolutional kernel of the convolutional layer and the weights in the neurons of the hidden layer of the fully-connected neural network to obtain a model with high classification accuracy.

**FIGURE 5.**
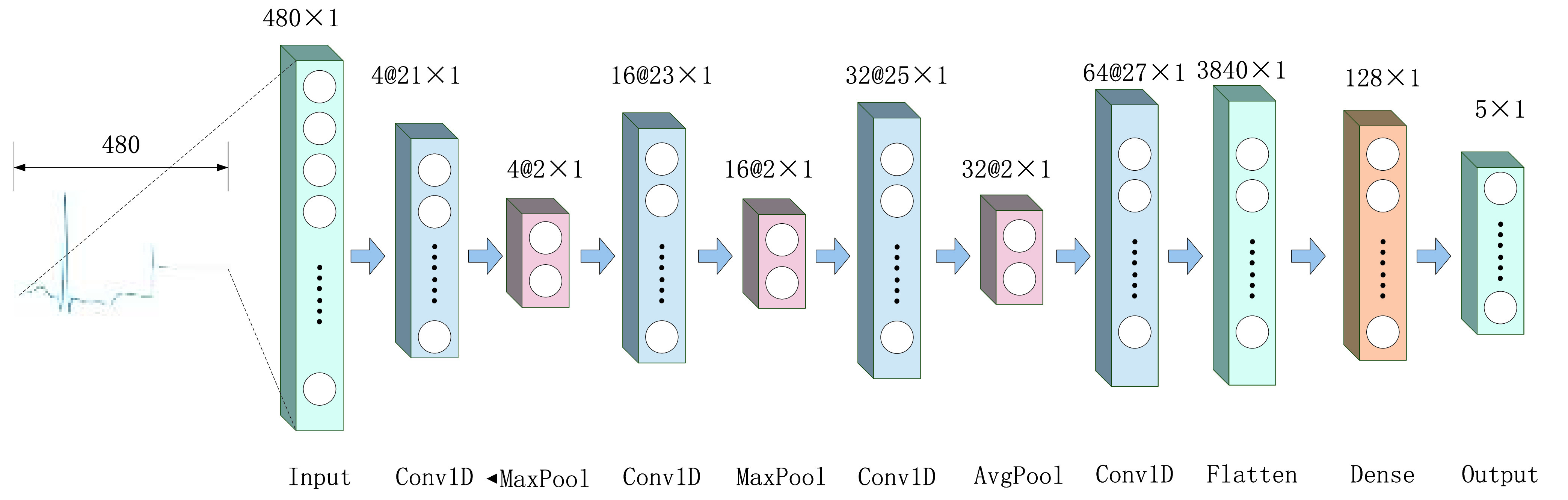
Model structure.

Among CNN techniques, 1D-CNN has good feature extraction for one-dimensional sequential data [51]. This makes 1D-CNN very suitable and being used for feature extraction of ECG signals. The 1D-CNN in this paper consists of an input layer, four convolutional layers, three pooling layers, a Flatten layer, a fully connected layer, and an output layer. The wider perceptual field can make the features of the response more holistic and global, and at the same time can optimize the feature extraction effect of 1D-CNN [52]. Therefore, this work sets a wider perceptual field in 1D-CNN. For the model input 300 temporal ECG signal data combined with 180 corresponding frequency domain information data into 480 data, and finally the classified results are output. The model structure is shown in Figure 5.

The first layer of the model is the input layer, which consists of 480 input nodes. The second layer is a one-dimensional convolutional layer, consisting of 4 channels of 21 $\times $ 1 convolutional kernels with a move step of 1 and a fill method of SAME to keep the dimensionality of the convolved data constant. The third layer is a maximum pooling layer with a pooling range of 2 $\times $ 1 and a move step of 2. The fourth layer is a one-dimensional convolutional layer consisting of 16 channels of 23 $\times $ 1 convolutional kernels with a move step of 1 and a fill method of SAME. The fifth layer is a maximum pooling layer with a pooling range of 2 $\times $ 1 and a move step of 2. The fourth layer is a one-dimensional convolutional layer consisting of 32 channels of 25 $\times $ 1 convolutional kernels with a move step of 1 and a fill method of SAME. The seventh layer is an average pooling layer with a pooling range of 2 $\times $ 1 and a shift step of 2. The eighth layer is a one-dimensional convolutional layer consisting of 64 channels of 27 $\times $ 1 convolutional kernels with a shift step of 1 and a fill step of SAME. The ninth layer is a Flatten layer, which stitches the convolutional results of the previous layer into a 3840 $\times $ 1 feature vector. The tenth layer is the Fully connected layer, which consists of 128 fully connected neurons, and the activation function of the neurons is RELU. the eleventh layer is the output layer, in which the function is softmax, which can output five kinds of results. The model properties of this study are shown in Table 1.

**TABLE 1:** Model Structure

Layer	Size	Strides	Output Shape
Input	(480,1)		(480,1)
Conv1D	(4,21,1)	1	(4,480,1)
MaxPooling	(4,2,1)	2	(4,240,1)
Conv1D	(16,23,1)	1	(16,240,1)
MaxPooling	(16,2,1)	2	(16,120,1)
Conv1D	(32,25,1)	1	(32,120,1)
AveragePooling	(32,2,1)	2	(32,60,1)
Conv1D	(64,27,1)	1	(64,60,1)
Flatten	(3840,1)		(3840,1)
Dense	(128,1)		(5,1)
Output	(5,1)		(5,1)

### E. Computational Complexity Analysis

The time complexity of performing FFT on the ECG signal is $O(N\cdot \log N)$. The computational complexity of the convolutional layer is $O\left({\sum \limits _{i=1}^{Q} {M_{i}} \cdot K_{i} \cdot C_{i-1} \cdot C_{i} }\right)$. Where Q is the number of convolutional layers. $M_{i} $ is the length of the feature vector output from the i-th layer convolutional kernel. $K_{i} $ is the length of the convolution kernel of the i-th layer. $C_{i} $ is the number of channels of the i-th layer convolution. The computational complexity of the fully connected layer is $O(I\cdot D\cdot U)$, I is the number of inputs, D is the number of intermediate layer neurons, and U is the number of outputs. The time complexity of this algorithm is $O\left({N\cdot \log N+I\cdot D\cdot U+\sum \limits _{i=1}^{Q} {M_{i}} \cdot K_{i} \cdot C_{i-1} \cdot C_{i} }\right)$.

## IV. Results and Analysis

The experimental hardware platform is a laptop computer configured with Intel Core i7-4720HQ 2.60GHz CPU, 16GB RAM memory and NVIDIA GeForce GTX 980M GPU. The experiments are curried on Windows 8.1 operating system using TensorFlow deep learning tool in Python 3.6 environment.

The study used five types of ECG data from the MIT-BIH arrhythmia dataset to train the model for the classification [53]. Those five types of heartbeat types are normal heartbeat (N), atrial premature beats (A), premature ventricular beats (V), left bundle branch block (L), and right bundle branch block (R). The database is from the Harvard-MIT Division of Health Sciences and Technology Biomedical Engineering Center. It contains 48 ECG signal data of about 30 minutes, and each heartbeat cycle is annotated in the annotation file of each data by a cardiologist The R-wave peak position and diagnostic category are marked in the annotation file of each data. During the model training and identification phase, the heartbeat can be localized according to the peak position of the heartbeat R-wave, and then a complete heartbeat cycle can be read into the model according to its position.

**FIGURE 6.**
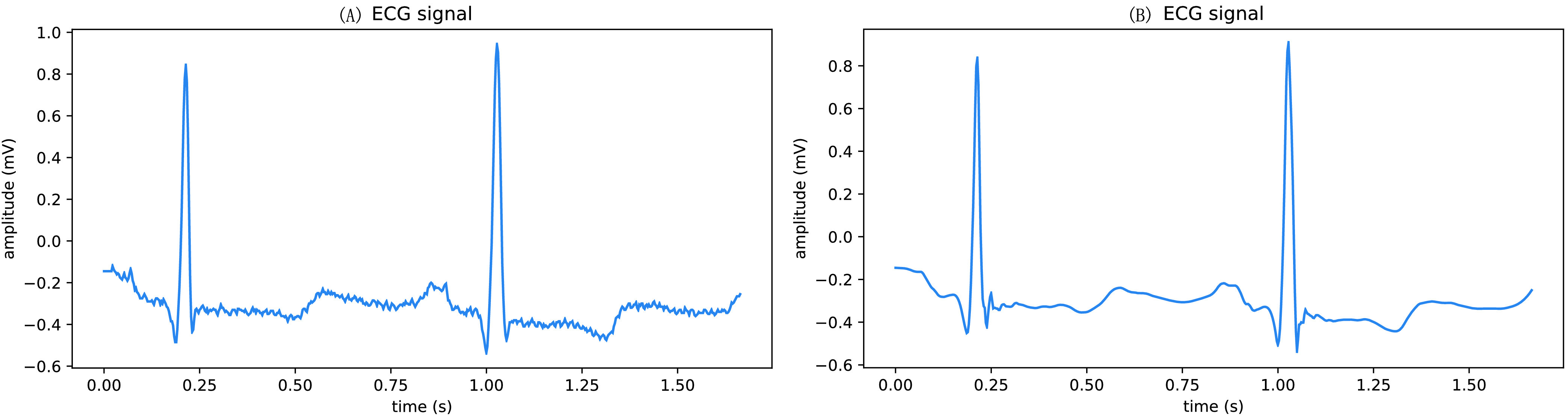
Filtering effect.

The experiments begin with filtering the ECG signal provided by the dataset. The ECG signal is decomposed on nine scales using a fifth-order Daubechies wavelet (db5) as the wavelet basis of the wavelet transform. After decomposition, the high-frequency components are mainly distributed in the D1 and D2 high-frequency components, and these two high-frequency components are set to zero to filter out this part of high-frequency noise. The other high-frequency components D3 to D9 are subjected to a soft threshold filtering operation. Finally, signal reconstruction of the processed individual classification is performed to complete the filtering of the ECG signal. The effect is shown in Figure 6, in which (A) is the ECG signal before filtering and (B) is the ECG signal after filtering.

In the model training process, the labeled data from the MIT-BIH arrhythmia dataset are used to directly obtain the location information of the R-wave peaks in the ECG signal, and each heartbeat cycle of the ECG signal is segmented by this location information. However, in practical applications, the R-wave positions of the ECG signal are not labeled, so the R-wave positions in the ECG signal need to be located before using the model in this study to classify the ECG signal in order to segment each individual heartbeat cycle.

**FIGURE 7.**
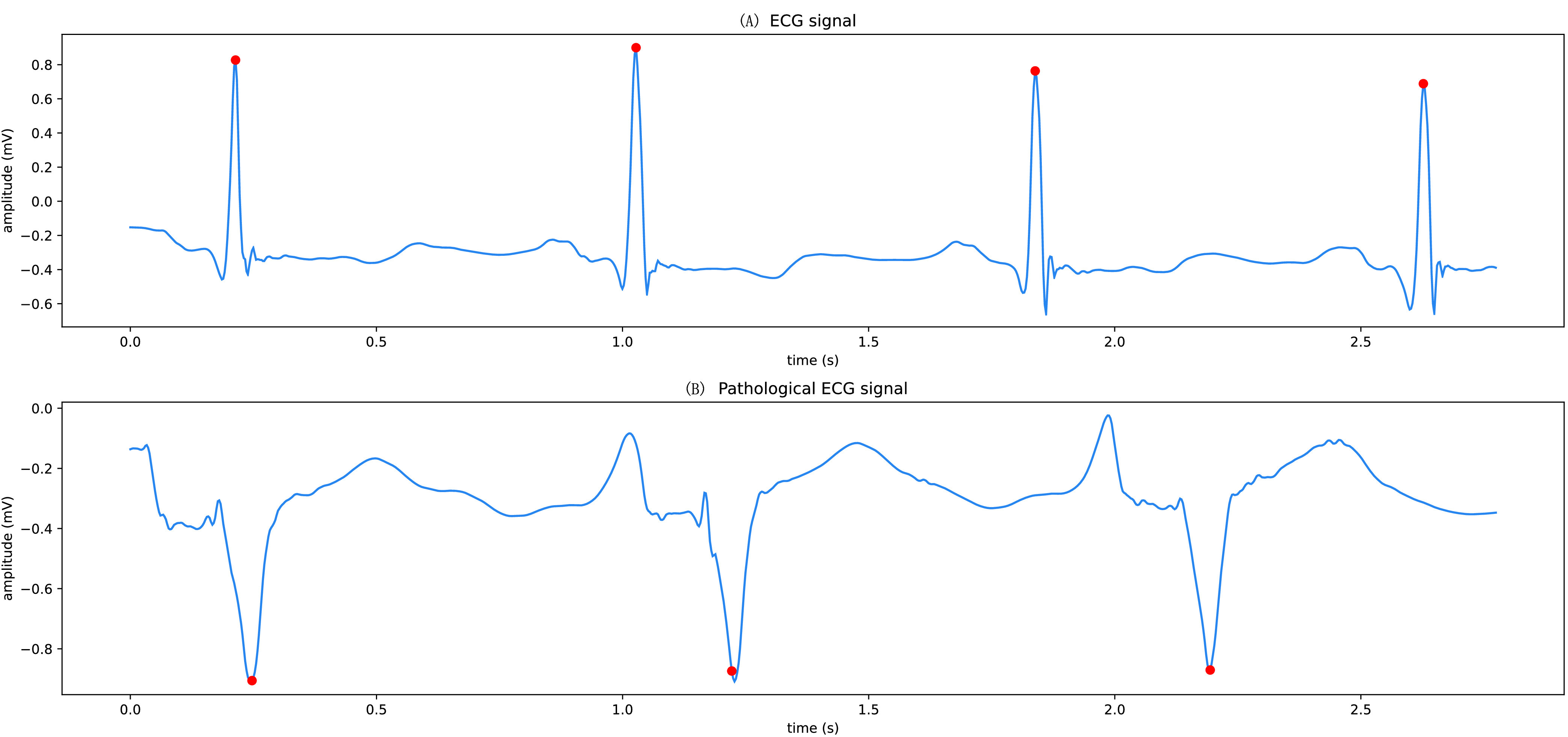
R-wave positioning effect.

The R-wave localization experiment uses the localization algorithm described above in this paper, in which the sliding window size J is set to 80, the sliding step size S is set to 30, the threshold of the difference between the maximum and minimum values within the reconstructed signal window is set to 0.15, the one-sided interval width K is set to 20, and the distance L is set to 50. Since the MIT-BIH arrhythmia dataset has a few small deviations from the R-wave peak position labeling, when the localization results are compared with the labeling results, the point is considered to be accurately localized as long as the difference between the two positions does not exceed 3. The accuracy of this method on normal ECG signals is extremely high, reaching 99.64% accuracy for R-wave localization on the healthy data set #100 of the MIT-BIH arrhythmia data set and 87.75% accuracy for R-wave localization on the diseased data #106. Although the localization results on the diseased data still need to be improved, the accuracy has been greatly improved compared to that of localization by first-order difference threshold. The R-wave localization effect is shown in Figure 7, where (A) is the R-wave localization effect of the normal ECG signal and (B) is the R-wave localization effect of the lesioned signal.

The positions of R-wave peaks of ECG signals are marked with red dots, which shows that the method is effective in locating the positions of R-wave peaks of various types of ECG signals.

**FIGURE 8.**
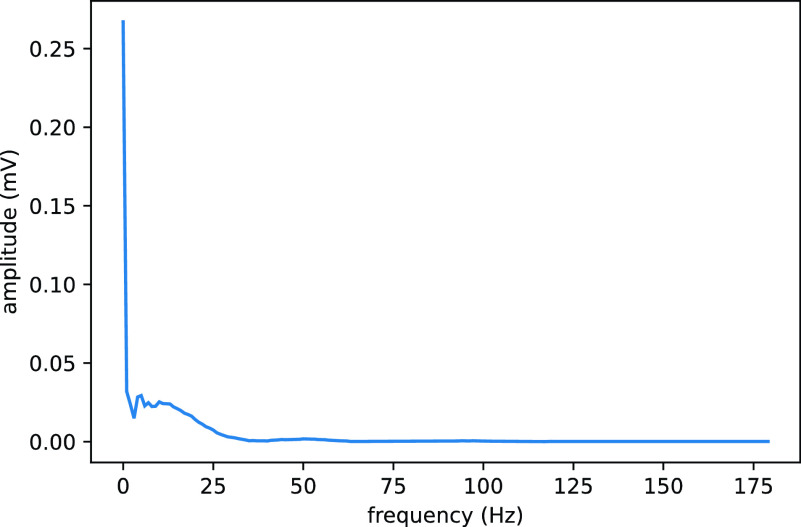
Heartbeat cycle spectrogram.

Since the sampling frequency of the data set used in this experiment is 360 Hz, the sampling length n is set to 100. Based on the R-wave localization results, the R-wave position with the first 99 and the next 200 data, a total of 300 data, is extracted as a heartbeat cycle signal to be identified. A fast Fourier transform with frequency set to 360Hz is performed on this signal to obtain the amplitude distribution of the signal in the frequency band from 0Hz to 180Hz, as shown in Figure 8.

A total of 48 records were available in the MIT-BIH arrhythmias database. In order to maintain lead consistency, MLII leads are usually used in studies. 102 and 104 did not have MLII lead data, so these two data were discarded and only 46 data were used. The R-wave crest position of each heartbeat in the record was labeled by the expert, and each heartbeat could be localized according to the R-wave crest position speak. In these 46 data, there are 99,188 heartbeat cycles. These 99188 ECG signals were divided, and 70% of these heartbeat cycles were used for model training, and the remaining 30% were used for model testing.

After the extraction of the frequency domain information of the ECG signal is completed, it is stitched with the ECG timing signal to form a length of 480 data for input into the diagnostic model for recognition. The epochs of this experimental training model are 30, and the loss function used is cross-entropy, whose mathematical expression is shown in equation (5).

**FIGURE 9.**
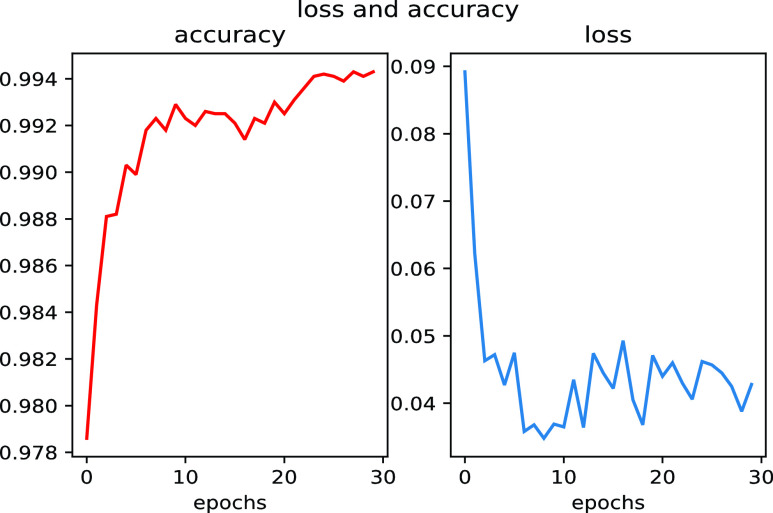
Changes in loss and accuracy.

\begin{equation*} L 1=-\frac {1}{N} \sum _{i=1}^{N} \sum _{c=1}^{5} y_{ic} \log \left ({p_{ic}}\right) \tag{5}\end{equation*}

where $y_{ic} $ denotes the true category of the sample, and $y_{ic} $ takes 1 if the category of sample $i$ is equal to c and 0 otherwise, $p_{ic} $ denotes the predicted probability that sample i belongs to category $c$, $N$ denotes the total number of samples, and $c=1$, 2, 3, 4, and 5 denote the five types of heart beats, namely normal beats (N), abnormal premature atrial beats (A), premature ventricular beats (V), left bundle branch block (L), and right bundle branch block (R), respectively. The electrical signal types. Figure 9 shows the changes in loss and accuracy during training.During the training process of the model, accuracy gradually rises to above 99% with the increase of training times, and the change curve of loss gradually converges.

The method in this study achieved an accuracy of 99.43% for the five diagnoses of ECG signals, indicating that the ECG diagnostic model possesses a high reliability. In Table 2, comparing this method with some methods of ECG signal classification in the last five years [34], [54], [55], [56], [57], the method of this paper using time-frequency domain fusion with 1D-CNN achieves higher accuracy.

**TABLE 2:** Comparison of Methods

Study	Method	Accuracy
J. Niu et al. [54]	Using symbolic representation and multi view convolution neural network	96.40%
Alquran H et al. [55]	Using CNN algorithm to evaluate high-order spectrum estimation, bispectrum and third-order cumulants of ECG signals	97.80 %
A.M.Shaker et al. [56]	Using generative adversarial networks and CNN	98.00%
Jun T J et al. [57]	Using 2D-CNN	99.05%
Yildirim Ö. et al. [34]	Using deep bidirectional LSTM network model	99.39%
Ours work	Using time-frequency domain fusion and 1D-CNN	99.43%

## V. Conclusion

In this paper, we propose a method to extract the frequency domain information of ECG signal and feed it into 1D-CNN for feature extraction and classification together with the original timing signal. The method achieves good results in identifying and classifying the ECG signals of each heartbeat cycle, and achieves 99.43% accuracy on the MIT-BIH arrhythmia dataset.

Despite the better results obtained by this method, there have some drawbacks. Since the extraction of frequency domain information relies on the division of individual heartbeat cycles. When the heartbeat in the ECG signal is divided incorrectly, it will cause the model to misdiagnose the heartbeat state. Moreover, the method is not able to diagnose a segment of ECG signal directly. It is necessary to first extract the complete heartbeat cycles from that segment of ECG signal, so that the extracted heartbeat cycles can be classified. Since the noise of the ECG signal affects the division of the heartbeat cycle, this leads to the requirement of the denoising quality of the ECG signal for this algorithm. Subsequent research will consider deploying the ECG diagnostic classification model of this experiment on a microcontroller and using it to make an offline ECG real-time diagnostic device.
